# The potential role of the osteopontin–osteocalcin–osteoprotegerin triad in the pathogenesis of prediabetes in humans

**DOI:** 10.1007/s00592-017-1065-z

**Published:** 2017-11-18

**Authors:** Giuseppe Daniele, Deidre Winnier, Andrea Mari, Jan Bruder, Marcel Fourcaudot, Zuo Pengou, Andrea Hansis-Diarte, Christopher Jenkinson, Devjit Tripathy, Franco Folli

**Affiliations:** 1 Department of Medicine, Division of Diabetes, University of Texas, Health Science Center at San Antonio, 7703 Floyd Curl Drive, San Antonio, TX 78229 USA; 2 Institute of Neuroscience, National Research Council, Padua, Italy; 3 Department of Medicine, Endocrine division, University of Texas, Health Science Center, San Antonio, TX USA; 4 Universita’ degli Studi di Milano, School of Medicine, Dipartimento di Scienze Della Salute, Milan, Italy; 5 Department of Medicine, Azienda Socio-Sanitaria Santi Paolo e Carlo, Via A. Di Rudini, 8, 20100 Milan, Italy

**Keywords:** Osteopontin, Osteocalcin, Osteoprotegerin, Leptin, Adiponectin, Prediabetes, Impaired glucose tolerance, Impaired fasting glucose, Impaired glucose regulation, Type 2 diabetes mellitus, Glucose metabolism, Euglycemic hyperinsulinemic clamp, Whole body insulin sensitivity, Beta-cell function, Human

## Abstract

**Aims:**

To examine the relationship between hormones involved in bone remodeling and glucose metabolism alterations in prediabetes.

**Methods:**

Individuals (*n* = 43) with NGT (BMI = 31.1 ± 1.1 kg/m^2^) and individuals (*n* = 79) with impaired glucose regulation (IGR) (BMI = 31.9 ± 1.2 kg/m^2^) including subjects with IFG, IGT, and IFG-IGT underwent OGTT and DXA. Osteopontin (OPN), osteocalcin (OCN), osteoprotegerin (OPG), and PTH levels were measured at fasting. Beta-cell function was calculated using C-peptide deconvolution. Dynamic indexes of insulin sensitivity were calculated from OGTT. A subgroup underwent to a euglycemic hyperinsulinemic clamp with 3-^3^H-glucose to estimate the endogenous glucose production (EGP) and insulin-mediated body glucose disposal (TGD/SSPI).

**Results:**

OPN was higher in IGR compared to NGT (5.3 ± 0.5 vs. 3.3 ± 0.2 μg/mL; *p* = 0.008) and in isolated IGT compared to IFG and IFG-IGT (6.3 ± 0.5 vs. 4.5 ± 0.3 and 5.4 ± 0.5 μg/mL; *p* = 0.02). OCN was similar in IFG and NGT but lower in IGT and IFG-IGT compared to NGT (7.2 ± 0.3 and 5.4 ± 0.2 vs. 8.3 ± 0.3 ng/mL; *p* < 0.01). OPN was positively correlated with HbA1c, fasting and 2 h plasma glucose and PTH. OCN was negatively correlated with body fat, 2 h plasma glucose, insulin and positively correlated with Stumvoll index. OPG correlated with TGD/SSPI (*r* = − 0.29; *p* < 0.05), EGP, and hepatic insulin resistance index in IGR (*r* = 0.51, *r* = 0.43; *p* < 0.01). There was no correlation between PTH and insulin sensitivity or Beta-cell function parameters.

**Conclusions:**

In prediabetes, hormones known to be involved in bone remodeling may affect glucose metabolism before overt T2DM occurs with tissue-specific mechanisms.

**Electronic supplementary material:**

The online version of this article (10.1007/s00592-017-1065-z) contains supplementary material, which is available to authorized users.

## Introduction

Type 2 diabetes mellitus (T2DM) is a progressive disease, preceded by a preclinical phase of impaired glucose regulation, in the fasting state and/or in the postprandial state (IFG, impaired fasting glucose, and IGT, impaired glucose tolerance) [1]. A number of alterations involving different tissues have been found to contribute to the development and progression of hyperglycemia, and more relevantly, the progressive alterations of insulin, glucagon, and somatostatin production and secretion from the endocrine pancreas and insulin action in skeletal muscle, liver, and adipose tissue are the known hallmarks of prediabetes and T2DM [2–6]. Besides the increased risk for T2DM, individuals with prediabetes may suffer from complications (e.g., nephropathy, retinopathy, neuropathy) traditionally considered to be complications of T2DM [7–9], which emphasize the importance of early diagnosis together with a more in-depth understanding of its pathogenic determinants. Bone has recently emerged as a new important organ in the regulation of glucose metabolism, which might cross talk with known physiologically relevant organs for glucose metabolism, by releasing hormones and cytokines such as osteocalcin and sclerostin, that are known to be critical regulators of bone mass through its remodeling [10, 11]. Osteocalcin (OCN) is an osteoblast-specific protein that is secreted in large amounts in the bone extracellular matrix. Beyond the effects on bone remodeling, OCN regulates Beta-cell proliferation, insulin gene expression, and secretion in both mice and humans suggesting a potential role in glucose metabolism and in the pathogenesis of glucose alterations in T2DM physiopathology [12, 13].

Also other cytokines, which are secreted by bone cells and are involved in bone remodeling, may also participate in glucose metabolism regulation in humans, and their alterations might be involved in the pathogenesis of prediabetes and T2DM.

Osteopontin (OPN), a multifunctional protein expressed in several different cell types including adipocytes, with the bone being a major production source, binds to integrins and CD44 receptors to recruit macrophages and T cells to inflammatory sites. OPN has been shown to be increased in T2DM [5], and it is probably implicated in subclinical inflammation [5], insulin resistance [14], obesity, and obesity-induced hepatic steatosis. Osteoprotegerin (OPG) is an inflammatory cytokine receptor implicated in bone remodeling. It is a soluble glycoprotein of the tumor necrosis factor receptor superfamily, responsible for osteoclastogenesis inhibition [15]. OPG is expressed in vivo by vascular smooth muscle cells, hepatocytes, and osteoblasts, and it might be an important factor to understand the relationship between bone mineralization and vascular pathology, i.e., atherosclerotic plaques calcification. Clinical studies have implicated OPG as a risk factor for progressive atherosclerotic cardiovascular disease [16, 17]. The adipose tissue-derived hormones leptin and adiponectin are independently and inversely associated with insulin resistance, glucose metabolism, and subclinical inflammation. However, they might also be involved in the bone–glucose metabolism cross talk because both adiponectin and leptin have a role in modulating bone resorption and osteoblasts differentiation and activation [18].

The aim of the present study is to investigate the relationship between bone-derived hormones potentially involved in the regulation of glucose metabolism (OPN, OCN, and OPG) by evaluating insulin sensitivity and beta-cell function in subjects with normal glucose tolerance and prediabetes, with OGTT and the euglycemic clamp.

## Research design and methods

The study population included 43 healthy normal glucose tolerant (NGT) individuals and 79 individuals with impaired glucose regulation (IGR). In this population group, we have previously studied the relationship between sclerostin and glucose homeostasis [11]. All subjects underwent an oral glucose (75 g) tolerance test (OGTT), dual-energy X-ray absorptiometry (DXA) whole body scan for body composition and determination of serum osteopontin, total osteocalcin, osteoprotegerin, parathyroid hormone (PTH), leptin, and adiponectin levels. A subgroup (*n* = 18 with NGT and *n* = 30 with IGR) underwent also a euglycemic hyperinsulinemic clamp in combination with tritiated glucose infusion, to directly assess whole body glucose uptake (which is primarily skeletal muscle glucose uptake) and hepatic glucose production in the fasting state and its suppression after insulin infusion (Supplementary Fig. 1). The study protocol was approved by the Institutional Review Board of the University of Texas Health Science Center and the South Texas Veterans Healthcare System, Audie Murphy Hospital at San Antonio, Texas. The study protocol was conducted in accordance with the guidelines of the Declaration of Helsinki. Written and oral informed consent was obtained from all participants enrolled in the study.

### Study procedures

All metabolic studies were carried out in the morning at the Bartter Research Unit of the South Texas Veterans Healthcare System, following a 10–12 h overnight fast.

1. OGTT: A catheter was placed in an antecubital vein, and blood samples were collected at − 30, − 15, 0, 30, 60, 90, and 120 min for determination of plasma glucose (PG), C-peptide, insulin, glucagon, and FFA concentrations.
2. Serum osteopontin, total osteocalcin, osteoprotegerin, PTH, leptin, and adiponectin levels were measured at fasting.
3. Hyperinsulinemic euglycemic clamp was performed as previously described [11] (Supplementary Fig. 2).
4. DXA: DXA whole body scan was performed to determine fat and lean body mass and whole body minus head bone mineral density (Hologic, Waltham, MA, USA).

### Calculations

Beta-cell function was assessed from the OGTT using a model describing the relationship between insulin secretion (ISR, expressed in pmol.min^−1^ m^−2^) and glucose concentration as the sum of two components. The Beta-cell glucose sensitivity, the rate sensitivity, and the potentiation factor were estimated from glucose and C-peptide concentrations (using C-peptide deconvolution) as previously described [19, 20]. Under steady-state post-absorptive conditions, the rate of endogenous glucose appearance (Ra) was calculated as the 3-^3^H-glucose infusion rate (DPM/min) divided by the steady-state plasma 3-^3^H-glucose specific activity (DPM/mg). During the euglycemic insulin clamp, the rate of glucose appearance (Ra) was calculated with the Steele’s equation, using a distribution volume of 250 mL/kg [21]. Endogenous (primarily reflecting hepatic) glucose production (EGP) was calculated by subtracting the exogenous glucose infusion rate from Ra. The rate of insulin-mediated total body glucose disposal (TGD/SSPI), hepatic insulin resistance index, and adipose tissue insulin resistance index (ATIRI) was calculated as previously described [11].

### Biochemical and hormonal analysis

Serum osteopontin, total osteocalcin, osteoprotegerin, PTH, adiponectin, and leptin levels were measured using the human-specific Milliplex map kit according to the manufacturer’s instructions in duplicate (Millipore, St Charles, MO, USA). The intra-assay and inter-assay variability was < 5%. Plasma glucose levels were measured using the glucose oxidase method (GM9; Analox Instruments, London, UK). Plasma insulin and C-peptide levels were measured by RIA method (Siemens Medical Solutions Diagnostics, Tarrytown, NY). Plasma 3-3H-glucose radioactivity was measured in Somogyi serum precipitates.

### Statistical analysis

Values were calculated as mean ± SEM or as median (interquartile range) for variables with a skewed distribution. The difference between means in NGT and IGR was compared with two-sided unpaired *t* test and repeated-measures ANOVA, with time and group as factors. In order to further explore the association between bone markers and glucose metabolism alterations, we also performed a subgroup analysis on IFG, IGT, and combined IFG/IGT subjects, using ANOVA and post hoc analysis. Variables that were not normally distributed were log-transformed before analysis. A *p* value of < 0.05 (two-tailed analysis) was considered to be statistically significant. Pearson correlation (normal distribution) and Spearman correlation analyses (non-normal distribution) were used to assess the correlations between serum bone makers and other continuous parameters, and we used partial correlations to correct the possible influence of age, sex, BMI, body fat. Multivariate analyses were performed to evaluate the contribution of bone markers in the association with insulin resistance, insulin sensitivity, and insulin clearance. Data were analyzed using SPSS 20 (Statistical Package for Social Sciences, Chicago, IL, USA).

## Results

### Baseline characteristic of the study population

The characteristics of the whole population studied are reported in Supplementary Table 1. The characteristics of subgroup subjects studied by the hyperinsulinemic euglycemic clamp are reported in the Supplementary Table 2. The two study groups were well matched in age, sex, BMI, fat and lean body mass, and bone density.

### Bone metabolism markers in NGT and IGR subjects at fasting

Osteopontin serum levels were higher in IGR as compared to NGT (5.3 ± 0.5 vs. 3.3 ± 0.2 μg/L; *p* = 0.008) (Table 1). However, when subjects were divided according to glucose tolerance status, osteopontin tended to be higher in isolated IGT and combined IFG-IGT subjects compared to NGT subjects being significantly higher in isolated IGT only (6.3 ± 0.5 vs. 3.3 ± 0.2 μg/L; *p* = 0.02). Total osteocalcin serum levels did not differ between NGT and IGR but when subjects were divided according to glucose tolerance status, total osteocalcin serum levels were significantly lower in combined IFG-IGT and isolated IGT subjects as compared to IFG and NGT subjects (5.4 ± 0.2 and 7.2 ± 0.3 vs. 8.0 ± 0.5 and 8.3 ± 0.3, *p* < 0.01 IFG-IGT vs. NGT and IFG; *p* < 0.01 IGT vs. NGT and IFG, respectively).

**Table 1:** Circulating levels of Osteopontin, Total Osteocalcin, Osteoprotegerin, PTH, Adiponectin and Leptin according to glucose tolerance status.

Characteristic (mean ± SEM)	NGT (*n* = 43)	IGR (*n* = 79)	IFG (*n* = 27)	IGT (*n* = 18)	IFG and IGT (*n* = 34)	*p* value
Osteopontin (μg/L)	3.3 ± 0.2	5.3 ± 0.5*	4.5 ± 0.3	6.3 ± 0.5^£^	5.4 ± 0.5^£^	< 0.05
Total osteocalcin (ng/mL)	8.3 ± 0.3	7.1 ± 0.4	8.0 ± 0.5	7.2 ± 0.3^£^	5.4 ± 0.2^£^	0.02
Osteoprotegerin (pg/mL)	458 ± 16	436 ± 18	443 ± 39	455 ± 54	421 ± 20	ns
PTH (pg/mL)	147 ± 8	147 ± 6	137 ± 10	141 ± 12	159 ± 13	ns
Adiponectin (μg/mL)	6362 ± 562	4835 ± 302*	4697 ± 426	4894 ± 606	4915 ± 541^£^	< 0.05
Leptin (ng/mL)	22.6 ± 2.7*	29.5 ± 2.7*	26.8 ± 3.9	24.8 ± 4.6	34.3 ± 4.1^£^	< 0.05

Characteristic (mean ± SEM)	NGT (*n* = 18)	IGR (*n* = 30)	IFG (*n* = 12)	IGT (*n* = 4)	IFG and IGT (*n* = 14)	*p* value*
Clamp subgroup						
Osteopontin (μg/L)	2.9 ± 0.4	4.8 ± 0.8*	5.5 ± 1.1	5.7 ± 1.9	3.9 ± 1.3^&^	0.04
Total osteocalcin (ng/mL)	7.0 ± 0.5	7.0 ± 0.7*	8.5 ± 1.5	7.9 ± 1.3	5.7 ± 0.6^£^	0.04
Osteoprotegerin (pg/mL)	480 ± 40	404 ± 18	433 ± 29	345 ± 76	396 ± 22	0.06
PTH (pg/mL)	131 ± 16	129 ± 9	135 ± 14	108 ± 7	129 ± 15	ns
Adiponectin (μg/mL)	6.3 ± 0.9	4.3 ± 0.5*	4.8 ± 0.7	6.5 ± 1.6	3.2 ± 0.5^&^	0.008
Leptin (ng/mL)	24.6 ± 4.4	28.7 ± 3.7	32.8 ± 7.5	22.6 ± 1.2	27.0 ± 3.8	ns

* *p* < 0.05 versus NGT

^£^ *p* < 0.05 versus IFG

^&^ *p* < 0.05 versus IFG and IGT

Adiponectin was significantly higher in NGT as compared to IGR, while leptin was significantly lower in NGT as compared to IGR.

Serum osteoprotegerin levels were not significantly higher in NGT compared to IGR, and PTH levels were similar between NGT and IGR. These levels did not differ across glucose tolerance status subgroups. When subjects were further divided according to sex or ethnicity, osteopontin, total osteocalcin, osteoprotegerin, and PTH levels were similar in males and females in both the NGT and IGR groups and no differences were found in subgroup analysis (data not shown). Adiponectin was higher in NGT as compared to IGR, while leptin was lower in NGT as compared to IGR.

### Relationships between bone metabolism markers and glucose metabolism

**Fig. 1:**
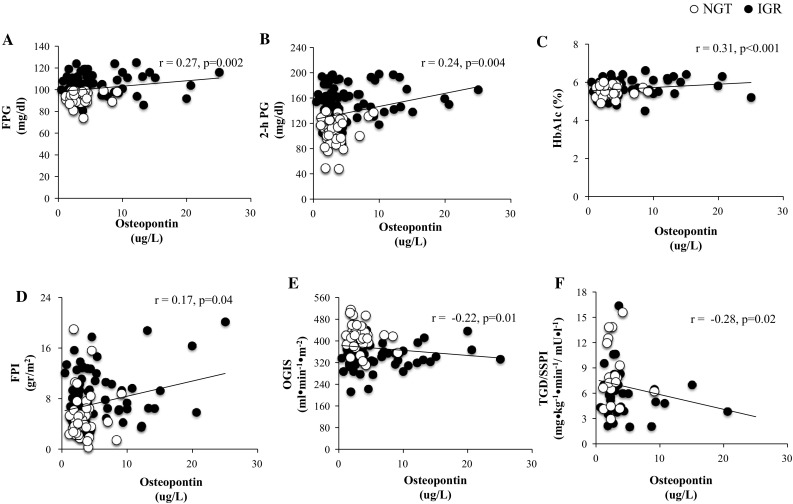
Partial correlatio between serum Osteopontin levels controlled for age, sex, BMI, body fat percentage and **a** Fasting Plasma Glucose (FPG), **b** 2-hours Plasma Glucose (2-hr PG), **c** HbA1c, **d** Fasting Plasma Insulin (FPI), **e** OGIS, **f** TGD/SPPI

Serum osteopontin levels were positively correlated with fasting glucose (*r* = 0.27; *p* = 0.002) (Fig. 1a), 2-hours plasma glucose (*r* = 0.24; *p* = 0.004) (Fig. 1b), HbA1c (*r* = 0.31; *p* < 0.001) (Fig. 1c), and fasting plasma insulin (*r* = 0.17; *p* = 0.04) (Fig. 1d). Moreover, osteopontin levels were negatively correlated with OGIS (*r* = − 0.22; *p* = 0.01) (Fig. 1e) and TGD/SSPI (*r* = − 0.28; *p* = 0.02) (Fig. 1f).

A multiple linear regression modeling was performed using 2 h plasma glucose as a dependent variable (Supplementary Table 3). In model 1, independent variables were body fat, BMI, age, and HbA1c. In model 2, osteopontin was added as independent variable to model 1 to assess the association of osteopontin with 2 h plasma glucose. In model 1, body fat was independently associated with 2 h plasma glucose (*r* ^2^ = 0.166; *p* = 0.001). In model 2, osteopontin was independently associated with 2 h plasma glucose and increased the *r* ^2^ as compared to model 1 (*r* ^2^ change: 0.032; *F* change: 4.019; *p* = 0.04).

**Fig. 2:**
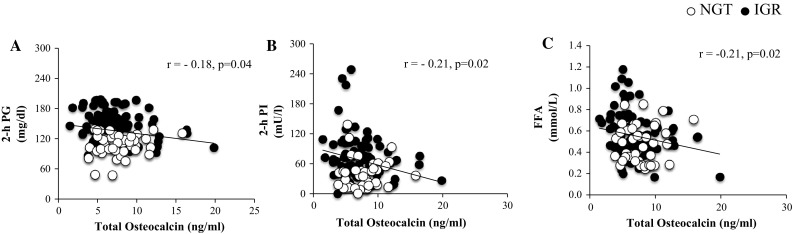
Partial correlation between serum Osteocalin levels controlled for age, sex, BMI, body fat percentage and **a** 2-hours Plasma Glucose (2-hr PG), **b** 2-hours Plasma Insulin (2-hr PI), **c** 2-hours Free Fatty Acids (FFA)

**Fig. 3:**
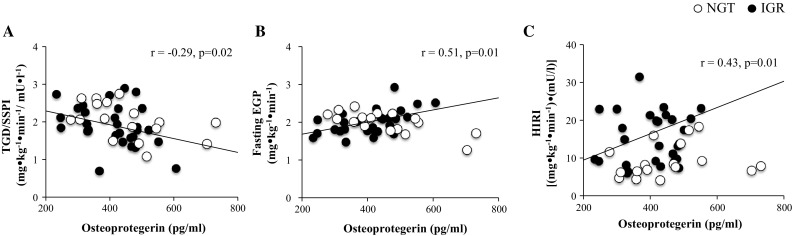
Partial correlation between serum Osteoprotegerin levels controlled for age, sex, BMI, body fat percentage and **a** TGD/SPPI, **b** Fasting EGP, **c** HIRI

Serum total osteocalcin levels were negatively correlated with 2-hours plasma glucose (*r* = − 0.19; *p* = 0.04) (Fig. 2a), 2-hours plasma insulin (*r* = − 0.21; *p* = 0.02) (Fig. 2b), and FFA (*r* = − 0.21; *p* = 0.02) (Fig. 2c), while no significant correlations were found between total osteocalcin and HbA1c, fasting plasma glucose and 2-hours plasma glucose. Circulating osteoprotegerin levels were inversely correlated with TGD/SSPI (*r* = − 0.29; *p* = 0.02 (Fig. 3a) and positively correlated with fasting EGP (*r* = 0.51; *p* = 0.01) (Fig. 3b) and HIRI (*r* = 0.43; *p* = 0.01) (Fig. 3c).

A multiple linear regression modeling was performed using fasting plasma glucose as dependent variable (Supplementary Table 3). In model 1, independent variables were body fat, BMI, age, and HbA1c. In model 2, osteoprotegerin was added as independent variable to model 1 to assess the association of osteoprotegerin with each dependent variable included in the model. In model 1, HbA1c was independently associated with fasting plasma glucose (*r* ^2^ = 0.175; *p* = 0.001). In model 2, osteoprotegerin was independently associated with fasting plasma glucose and increased the *r* ^2^ as compared to model 1 (*r* ^2^ change: 0.057; *F* change: 7.551; *p* = 0.007). Similarly, HbA1c was separately used as dependent variable (model 1). As expected, in model 1, fasting plasma glucose was independently associated with HbA1c (*r* ^2^ = 0.190). In model 2, osteoprotegerin was independently associated with HbA1c and increased the *r* ^2^ (*r* ^2^ change: 0.032; *F* change: 4.160; *p* = 0.044).

A multiple linear regression analysis was performed using fasting EGP as a dependent variable. In model 1, independent variables were body fat, BMI, fasting plasma glucose, fasting plasma insulin, age, and HbA1c. In model 2, osteoprotegerin was added as independent variable to model 1 to assess the contribution of osteoprotegerin to the association with fasting EGP. BMI was independent predictor of fasting EGP (*r* ^2^ = 0.534). Consistently, in model 2, osteoprotegerin was independently associated with fasting EGP and increased the *r* ^2^ (*r* ^2^ change: 0.145; *F* change: 5.901; *p* = 0.025).

PTH levels were not correlated with any of the glucose metabolism parameters examined.

There was no significant correlation between osteopontin, osteocalcin, osteoprotegerin, and PTH with beta-cell function parameters (i.e., fasting and total insulin secretory rates, glucose sensitivity, rate sensitivity, and potentiation factor) (Supplementary Table 4), ATIRI and insulin clearance at baseline and during OGTT (data not shown).

### Relationship between bone metabolism markers and adipocytokines with bone density and body composition

**Fig. 4:**
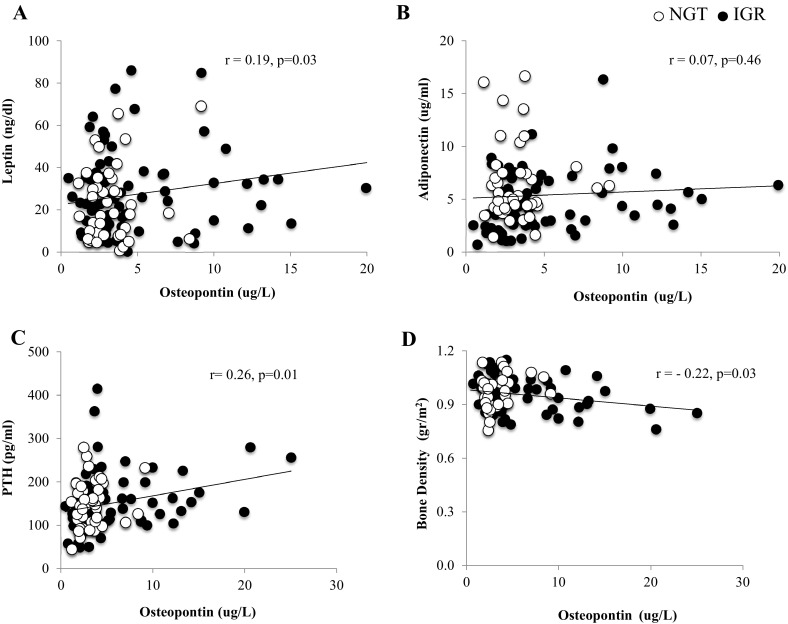
Partial correlation between serum Osteopontin levels controlled for age, sex, BMI, body fat percentage and **a** Leptin, **b** Adiponectin, **c** PTH, **d** Bone Density

Circulating osteopontin levels were positively correlated with leptin (*r* = 0.19; *p* = 0.03) (Fig. 4a) but not with adiponectin (*r* = 0.07; *p* = 0.46) (Fig. 4b). Moreover, osteopontin was positively correlated with PTH (*r* = 0.26; *p* = 0.01) and inversely with bone density (*r* = − 0.22, *p* = 0.03) (Fig. 4c, d).

**Fig. 5:**
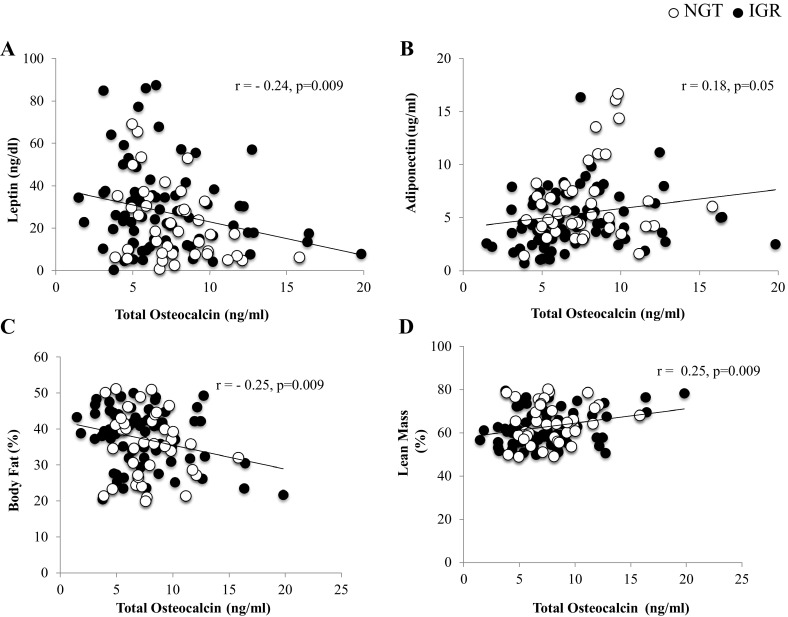
Partial correlation between serum Osteocalcin levels controlled for age, sex, BMI, body fat percentage and **a** Leptin, **b** Adiponectin, **c** Body Fat, **d** Lean Mass

Total Osteocalcin levels were negatively correlated with leptin (*r* = − 0.24; *p* = 0.009) (Fig. 5a) and positively with adiponectin although it was not significant (*r* = 0.18; *p* = 0.05) (Fig. 5b). Total osteocalcin levels were also inversely correlated with body fat (*r* = − 0.25; *p* = 0.009) and positively correlated with lean mass (*r* = 0.25; *p* = 0.009) (Fig. 5c, d).

## Discussion

This study demonstrates that circulating OPN levels increase as the glucose tolerance deteriorates in humans. This evidence is supported by the inverse and significant relationship between OPN levels and plasma glucose profiles and insulin sensitivity, suggesting a potential combined role in the glucose homeostasis abnormalities underlying prediabetes. We and others have shown that OPN levels are increased in type 1 and type 2 diabetes [5, 22] and in obesity and correlate with insulin resistance and hyperglycemia. Also increased circulating OPN is a marker of early coronary arteries calcification in type 2 diabetes [23] and a strong predictor of incipient diabetic nephropathy and all-cause mortality in type 1 diabetes [23]. OPN is associated with a subclinical inflammatory status, hyperglycemia, and insulin resistance in T2DM, and the present results suggest that it may exert its pro-inflammatory effect before the onset of T2DM, in its preclinical stage, i.e., IGR [5]. Conversely, total OCN levels decrease in IGR as compared to NGT in particular in subjects with both IFG and IGT. Several studies have reported low OCN levels in patients with diabetes and metabolic syndrome and the correlations between OCN and parameters of glucose metabolism have largely been consistent with animal models [23]. Also OCN levels were lower in diabetic compared to non-diabetic patients with metabolic syndrome [24]. Moreover, OCN levels were inversely associated with plasma glucose levels and fat mass in type 2 diabetes, and our study confirms the finding in prediabetes. Interestingly, we find a positive correlation between lean mass and OCN levels implying that OCN might be possibly involved in the modulation of skeletal muscle trophism, which is also impaired in T2DM. This hypothesis is supported by recent evidences that demonstrated that OCN receptors are expressed in mouse muscle and exogenous OCN administration augments insulin-stimulated skeletal muscle glucose uptake in C2C12 myotubes favoring the translocation of GLUT4 to the plasma membrane and following ex vivo muscle contraction [25]. Osteocalcin is also directly correlated with adiponectin, another important insulin-sensitizing hormone [26]. Moreover, OCN favors FA catabolism in myofibers. In animal studies, exogenous OCN increases the cellular energy sensor AMPK phosphorylation which promotes FA utilization in muscle by increasing the activity of CPT1B, a transporter of long-chain FAs into the mitochondria. On the other hand, OCN is inversely correlated with leptin and body fat mass, suggesting another potential aspect of body composition by which OCN might be finally involved in the regulation of glucose metabolism [25, 27].

Our results demonstrate that OCN levels are significantly and inversely correlated with circulating FFA concentration independently of BMI and insulin resistance, suggesting that it may it might be involved in the modulation of FFA utilization. On the other hand, non-alcoholic fatty liver disease (NAFLD) is strongly associated with insulin resistance [28] and a large portion of NAFLD patients have postprandial hyperinsulinaemia and abnormal glucose tolerance. As steatosis leads to decreased hepatic insulin sensitivity and increased gluconeogenesis, it has been hypothesized that NAFLD precedes the development of T2DM [29].

Primary NAFLD was a strong risk factor for impaired glycemic control at the population level independently of a wide range of confounders including insulin, adipocytokines, metabolic syndrome, physical activity, weight change during follow-up, and dietary intake. Recent evidences demonstrated that OPG levels were positively associated with visceral adipose tissue, HOMA-IR, adipokines arterial stiffness, and the number of atherosclerotic sites [30]. More interestingly, OPG was also associated with liver steatosis markers and liver fat content, suggesting a possible role of liver steatosis in the overproduction of OPG in metabolic patients [30]. In our study, high OPG circulating levels are associated with higher endogenous glucose production (primarily reflecting liver glucose production) and hepatic insulin resistance in IRG. These findings support the possibility that OPG might play a role in glucose homeostasis alterations which usually precede overt T2DM. Moreover, adipose tissue may have a direct role in metabolic alterations underlying prediabetes modulating the bone markers potentially affecting the glucose metabolism. Leptin activates β-2 adrenergic receptors on osteoblasts through the sympathetic nervous system and thereby decreases the differentiation and activation of osteoblasts while increasing the activity of osteoclasts in bone resorption. Hence, increased leptin is detrimental to bone formation while increasing bone resorption. Conversely, adiponectin stimulates the differentiation and mineralization of the osteoblast, while inhibiting osteoclast activity and bone resorption [31]. Moreover, several evidences demonstrated that both leptin and adiponectin can directly affect glucose metabolism [26, 32]. In the present study, both OPN and OCN are significantly correlated with leptin and adiponectin, suggesting that the modulation of glucose metabolism may be also interconnected and mediated by their effects on bone metabolism; therefore, further studies are needed to explore the molecular mechanisms underlying this cross talk. Moreover, it has been shown that excess PTH in subjects with chronic renal failure plays a major role in the genesis of the glucose intolerance of uremia [33]. Our study has some limitations. The cross-sectional design does not allow the study of potential changes in bone markers levels in the natural history of IGR, thus possibly providing a stronger link between glucose metabolism alterations and bone markers levels. Calcium and 25(OH) vitamin D have a role in the regulation of glucose metabolism [34–36]; however, calcium and 25(OH) vitamin D status has not been evaluated in this study. Future studies should evaluate the relation between bone-derived hormones, including sclerostin, osteopontin, osteoclastin, and osteoprotegerin with 25(OH) vitamin D, given their role in the regulation of glucose metabolism and insulin secretion, in humans. The sample size might affect the statistical power, although the study groups were well matched for age, sex, BMI, and body composition, including bone density. Correlations do not prove “cause and effect,” and additional factors may modulate the relationship between bone markers and glucose metabolism. Strengths of our study are the evaluation of circulating serum bone markers in subjects with NGT and IGR combined with the extensive and detailed evaluation of biochemical and clinical parameters of glucose metabolism, derived from OGTT and the gold-standard, euglycemic clamp with glucose tracer to evaluate hepatic glucose production. Also the results of this study add evidences on support of a complex inter-organ communication since bone is increasingly being recognized as an endocrine tissue that participates in regulating whole body fuel metabolism and glucoregulation. Moreover, it has been shown that bone mass represents a remarkable percentage of body weight. It should be noted that dual-energy X-ray absorptiometry (DXA) is based on the chemical model and does not provide a reliable measure of bone mass of an individual, since a direct method measuring the bone weight through dissection may be well-founded. The most important study on this topic is the Belgium study that demonstrated that the adipose tissue-free weight of bone is around 20% in men and 21% in women [37] and these percentages were similar to percentages observed in non-human primates [38].

This and previous studies in humans as well as mechanistic studies in cell and rodent models suggest that bone might represent a new important player in the regulation of glucose metabolism in aging and type 2 diabetes mellitus [5, 11, 39–41]. In conclusion, we believe that future studies should address the possibility that new drugs might be designed to target these novel markers of bone homeostasis as well as glucose metabolism in the elderly population, which is frail and more prone to type 2 diabetes and osteoporosis and related complications [42, 43].

## Electronic supplementary material

Below is the link to the electronic supplementary material.

Supplementary material 1 (PPTX 53 kb)

Supplementary material 2 (PPTX 61 kb)

Supplementary material 3 (DOCX 94 kb)

Supplementary material 4 (DOCX 72 kb)

Supplementary material 5 (DOCX 94 kb)

Supplementary material 6 (PPTX 51 kb)

## Abbreviations

T2DM: Type 2 diabetes Mellitus
DXA: Dual-energy X-ray absorptiometry
FPG: Fasting plasma glucose
PG: Plasma glucose
1-hPG: 1 h plasma glucose
2-hPG: 2 h plasma glucose
IR: Insulin resistance
IGR: Impaired glucose regulation
IGT: Impaired glucose tolerance
IFG: Impaired fasting glucose
OPN: Osteopontin
OCN: Osteocalcin
OPG: Osteoprotegerin
PTH: Parathyroid hormone
