# Supplementary material for: The potential role of the osteopontin–osteocalcin–osteoprotegerin triad in the pathogenesis of prediabetes in humans
Source: Acta Diabetol. 2017 Nov 18;55(2):139–48. doi: 10.1007/s00592-017-1065-z (PMC5816090; doi:10.1007/s00592-017-1065-z)
Supplement: Supplementary file 1 — Supplementary material 1 (PPTX 53 kb) [file 592_2017_1065_MOESM1_ESM.pptx]

## Slide 1
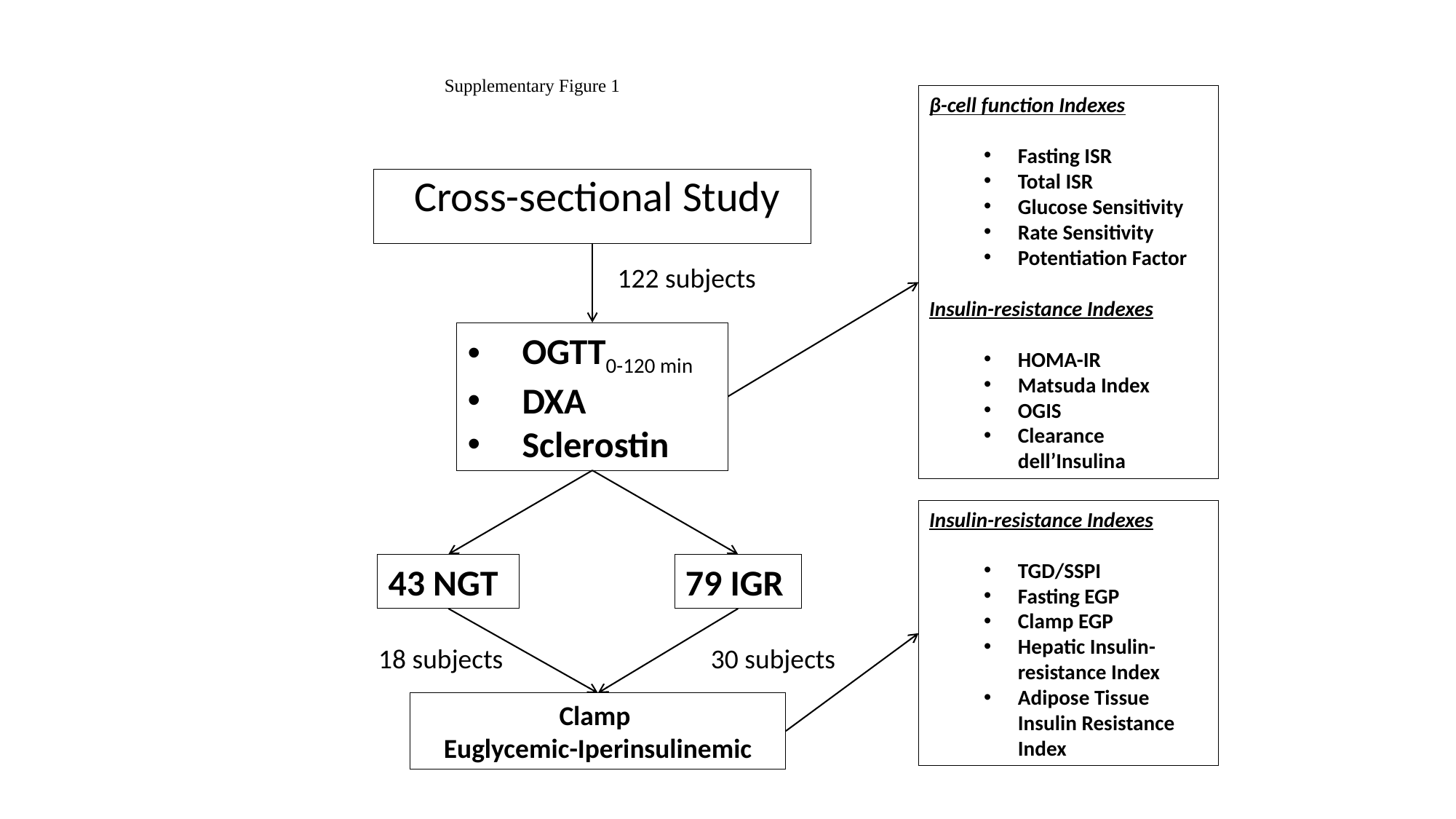

Supplementary Figure 1
β-cell function Indexes
Fasting ISR
Total ISR
Glucose Sensitivity
Rate Sensitivity
Potentiation Factor
Insulin-resistance Indexes
HOMA-IR
Matsuda Index
OGIS
Clearance dell’Insulina
 Cross-sectional Study
122 subjects
OGTT0-120 min
DXA
Sclerostin
Insulin-resistance Indexes
TGD/SSPI
Fasting EGP
Clamp EGP
Hepatic Insulin-resistance Index
Adipose Tissue Insulin Resistance Index
43 NGT
79 IGR
18 subjects
30 subjects
Clamp
Euglycemic-Iperinsulinemic
