# Supplementary material for: The potential role of the osteopontin–osteocalcin–osteoprotegerin triad in the pathogenesis of prediabetes in humans
Source: Acta Diabetol. 2017 Nov 18;55(2):139–48. doi: 10.1007/s00592-017-1065-z (PMC5816090; doi:10.1007/s00592-017-1065-z)
Supplement: Supplementary file 2 — Supplementary material 2 (PPTX 61 kb) [file 592_2017_1065_MOESM2_ESM.pptx]

## Slide 1
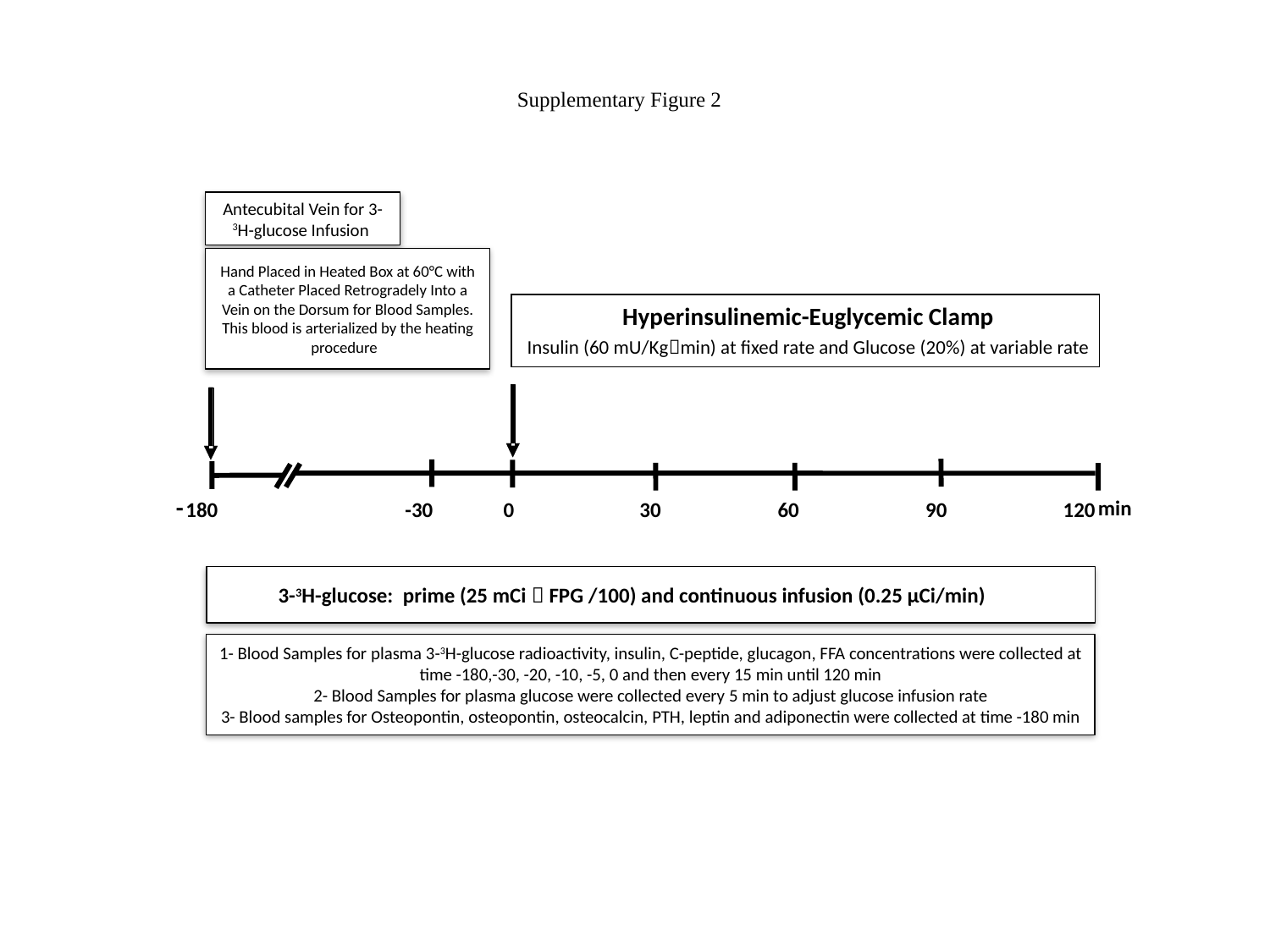

Supplementary Figure 2
Antecubital Vein for 3-3H-glucose Infusion
Hand Placed in Heated Box at 60°C with a Catheter Placed Retrogradely Into a Vein on the Dorsum for Blood Samples.
This blood is arterialized by the heating procedure
Hyperinsulinemic-Euglycemic Clamp
Insulin (60 mU/Kgmin) at fixed rate and Glucose (20%) at variable rate
-
min
180
-30
0
30
60
90
120
3-3H-glucose: prime (25 mCi  FPG /100) and continuous infusion (0.25 μCi/min)0.25
1- Blood Samples for plasma 3-3H-glucose radioactivity, insulin, C-peptide, glucagon, FFA concentrations were collected at time -180,-30, -20, -10, -5, 0 and then every 15 min until 120 min
2- Blood Samples for plasma glucose were collected every 5 min to adjust glucose infusion rate
3- Blood samples for Osteopontin, osteopontin, osteocalcin, PTH, leptin and adiponectin were collected at time -180 min
