# Supplementary material for: The potential role of the osteopontin–osteocalcin–osteoprotegerin triad in the pathogenesis of prediabetes in humans
Source: Acta Diabetol. 2017 Nov 18;55(2):139–48. doi: 10.1007/s00592-017-1065-z (PMC5816090; doi:10.1007/s00592-017-1065-z)
Supplement: Supplementary file 3 — Supplementary material 3 (DOCX 94 kb) [file 592_2017_1065_MOESM3_ESM.docx]

| Characteristic | mean±SEM  (*All subjects*) | Range  (min-max) |
| --- | --- | --- |
| Age (years) | 45.4±1.1 | 18-68 |
| Sex (Men/ Women) | 32/88 | - |
| BMI (kg/m^2^) | 31.6±0.6 | 18-53 |
| Fat content (%) | 37.2±0.8 | 20-51 |
| Lean Mass (%) | 62.8±0.8 | 49-80 |
| Bone Density (g/m^2^) | 0.96±0.09 | 0.76-1.15 |
| FPG (mg/dl) | 100±0.9 | 74-125 |
| 2-h PG (mg/dl) | 135±3.0 | 48-198 |
| HbA1c (%) (mmol/mol) | 5.60±0.04 (38±0.4) | 4.5-6.6 |
| FPI (mU/l) | 6.9 ±0.4 | 0.3-31.2 |
| FFA (mmol/l) | 0.55±0.02 | 0.27-1.18 |
| OGIS (ml•min^-1^•m^-2^) | 375±5 | 213-621 |
| HOMA-IR (mmol/l •mU/l) | 1.8±0.1 | 0.06-7.94 |
| Fasting Insulin Clearance (L•min^-1^•m^-2^) | 3.9±0.3 | 0.67-31.01 |
| OGTT Insulin Clearance (L•min^-1^•m^-2^) | 1.9±0.1 | 0.29-18.80 |
| Adipose Tissue Insulin Resistance Index (mmol/l • mU/l) | 3.8±0.3 | 0.01-17.38 |
| Fasting ISR (pmol/m^2^/min) | 123±4 | 36-249 |
| Total ISR (nmol/m^2^) | 65±2 | 23-135 |
| β-GS (pmol/m^2^/min/mmol) | 153±10 | 16-670 |
| Potentiation Factor (ratio) | 1.5±0.1 | 0.4-3.8 |
| Rate sensitivity (pmol/m^2^/mmol) | 1577±162 | 0.1-12658 |
| Osteopontin (μg/L) | 4.6±0.4 | 0.5-25.0 |
| Total Osteocalcin (ng/ml) | 7.4±0.3 | 0.1-19.8 |
| Osteoprotegerin (pg/ml) | 444±16 | 194-1101 |
| PTH (pg/ml) | 147±6 | 44-415 |
| Adiponectin (μg/ml) | 5.4±0.3 | 0.7-16.7 |
| Leptin (ng/ml) | 27.1±1.9 | 0.3-101.3 |

Supplementary Table 1
