# Supplementary material for: The potential role of the osteopontin–osteocalcin–osteoprotegerin triad in the pathogenesis of prediabetes in humans
Source: Acta Diabetol. 2017 Nov 18;55(2):139–48. doi: 10.1007/s00592-017-1065-z (PMC5816090; doi:10.1007/s00592-017-1065-z)
Supplement: Supplementary file 4 — Supplementary material 4 (DOCX 72 kb) [file 592_2017_1065_MOESM4_ESM.docx]

Supplementary Table 2

| Characteristic | mean±SEM  (*Clamp subjects*) | Range  (min-max) |
| --- | --- | --- |
| Age (years) | 45.6±1.8 | 18-68 |
| Sex (Men/ Women) | 11/37 | - |
| BMI (kg/m^2^) | 32.6±1.0 | 22-50 |
| Fat content (%) | 36.9±1.3 | 21-51 |
| Lean Mass (%) | 63.1±1.3 | 49-79 |
| Bone Density (g/m^2^) | 0.96±0.09 | 0.76-1.15 |
| TGD/SSPI (mg•kg^-1^•min^-1^/ mU•l^-1^) | 7.6±0.4 | 2.9-14.9 |
| Fasting EGP (mg•kg^-1^•min^-1^) | 2.02±0.04 | 1.27-2.93 |
| Clamp EGP (mg•kg^-1^•min^-1^) | 0.82±0.31 | 0.01-12.04 |
| Hepatic Insulin Resistance Index  [(mg•kg^-1^•min^-1^)• (mU/l)] | 12.9±0.9 | 4.2-26.0 |
| Osteopontin (μg/L) | 4.1±0.5 | 0.7-21.6 |
| Total Osteocalcin (ng/ml) | 7.0±0.5 | 3.1-19.8 |
| Osteoprotegerin (pg/ml) | 432±19 | 196-928 |
| PTH (pg/ml) | 129±7 | 44-279 |
| Adiponectin (μg/ml) | 5.0±0.5 | 0.7-16.1 |
| Leptin (ng/ml) | 27.1±2.9 | 4.3-84.6 |
