# Supplementary material for: The potential role of the osteopontin–osteocalcin–osteoprotegerin triad in the pathogenesis of prediabetes in humans
Source: Acta Diabetol. 2017 Nov 18;55(2):139–48. doi: 10.1007/s00592-017-1065-z (PMC5816090; doi:10.1007/s00592-017-1065-z)
Supplement: Supplementary file 5 — Supplementary material 5 (DOCX 94 kb) [file 592_2017_1065_MOESM5_ESM.docx]

| Multivariate Analysis | r^2^  (p value) | r^2^ change | F change  (p value) | F  (p value) | β | p value |
| --- | --- | --- | --- | --- | --- | --- |
| 2 Hours Plasma Glucose (mg/dl)  (*Dependent Variable*)  *Independent Variables* (Model 1) | 0.166  (0.001) | 0.166 | 5.125  (0.001) | 5.024  (0.001) |  |  |
| Body Fat (%)  BMI (Kg/m^2^)  Age (years)  HbA1c (%) |  |  |  |  | 0.193  0.084  0.018  0.327 | 0.037  0.353  0.842  0.000 |
| *Independent Variable* (Model 2)  Osteopontin (μg/L) | 0.198  (0.040) | 0.032 | 4.019  (0.040) | 5.024  (<0.001) | 0.183 | 0.040 |
| Fasting Plasma Glucose (mg/dl)  (*Dependent Variable*)  *Independent Variables* (Model 1) | 0.175  (0.001) | 0.175 | 5.445  (0.001) | 5.445  (0.001) |  |  |
| Body Fat (%)  BMI (Kg/m^2^)  Age (years)  HbA1c (%) |  |  |  |  | 0.039  0.135  0.038  0.373 | 0.667  0.136  0.679  0.000 |
| *Independent Variable* (Model 2)  Osteoprotegerin (pg/ml) | 0.231  (0.007) | 0.057 | 7.551  (0.007) | 6.143  (0.007) | 0.244 | 0.007 |
| HbA1c (%)  (*Dependent Variable*)  *Independent Variables* (Model 1) | 0.190  (<0.001) | 0.190 | 6.059  (<0.001) | 6.059  (<0.001) |  |  |
| Body Fat (%)  BMI (Kg/m^2^)  FPG (mg/dl)  2-h PG (mg/dl) |  |  |  |  | -0.160  0.020  0.292  0.218 | 0.861  0.827  0.004  0.034 |
| *Independent Variable* (Model 2)  Osteoprotegerin (pg/ml) | 0.577  (<0.001) | 0.032 | 4.160 (0.044) | 6.830  (<0.001) | 0.186 | 0.044 |
| Fasting EGP (mg•kg^-1^•min^-1^)  (*Dependent Variable*)  *Independent Variables* (Model 1) | 0.389  (0.096) | 0.389 | 2.119  (0.096) | 2.119  (0.096) |  |  |
| Body Fat (%)  BMI (Kg/m^2^)  FPG (mg/dl)  FPI (mU/l)  Age (Years)  HbA1c (%) |  |  |  |  | -0.128  -0.465  -0.258  0.205  -0.273  0.176 | 0.515  0.043  0.232  0.267  0.174  0.449 |
| *Independent Variable* (Model 2)  Osteoprotegerin (pg/ml) | 0.534  (0.025) | 0.145 | 5.901  (0.025) | 2.119  (0.096) | 0.544 | 0.025 |

Supplementary Table 3
