# Supplementary material for: The potential role of the osteopontin–osteocalcin–osteoprotegerin triad in the pathogenesis of prediabetes in humans
Source: Acta Diabetol. 2017 Nov 18;55(2):139–48. doi: 10.1007/s00592-017-1065-z (PMC5816090; doi:10.1007/s00592-017-1065-z)
Supplement: Supplementary file 6 — Supplementary material 6 (PPTX 51 kb) [file 592_2017_1065_MOESM6_ESM.pptx]

## Slide 1
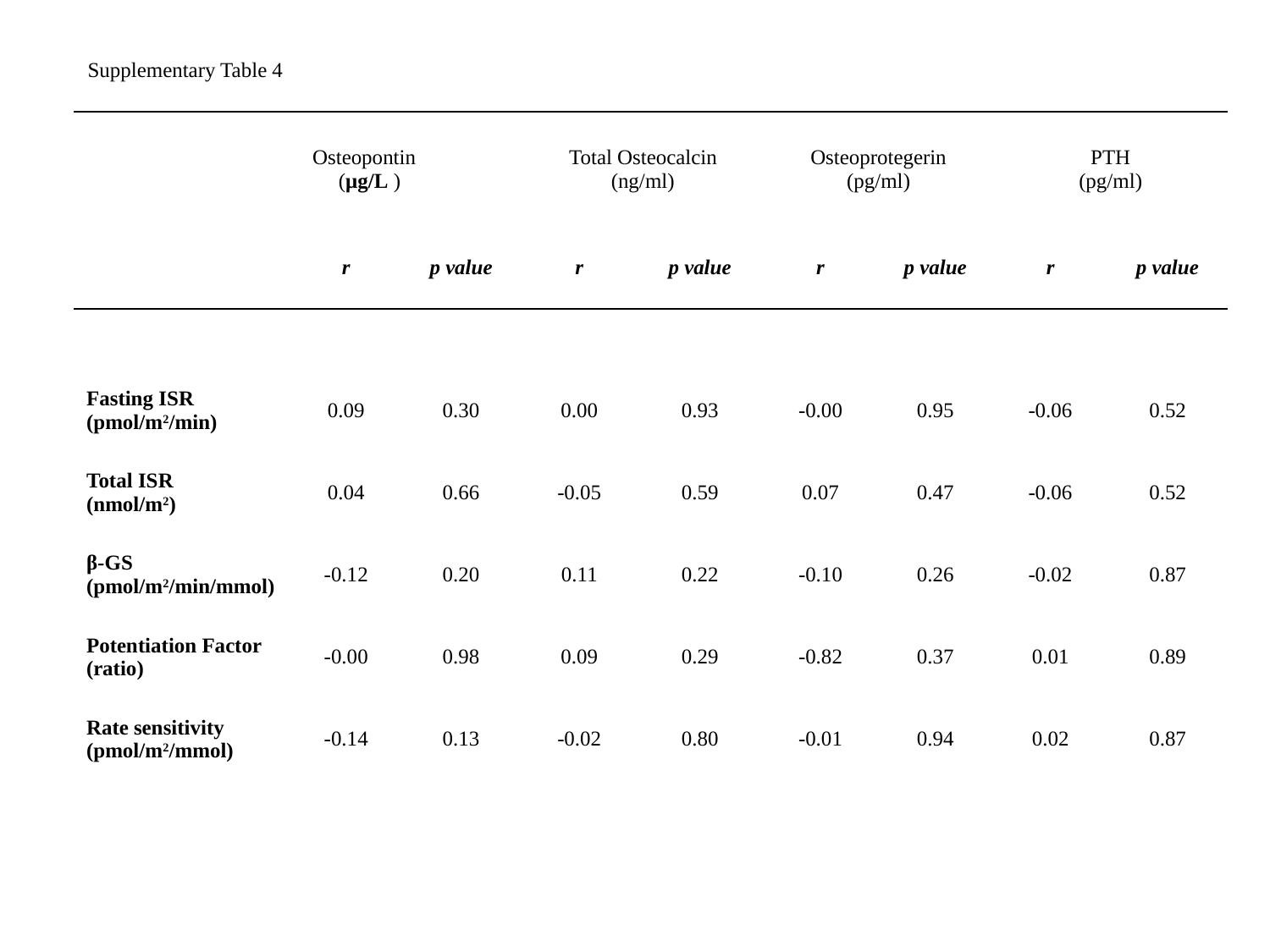

Supplementary Table 4
| Osteopontin (μg/L ) | | | Total Osteocalcin (ng/ml) | | Osteoprotegerin (pg/ml) | | PTH (pg/ml) | |
| --- | --- | --- | --- | --- | --- | --- | --- | --- |
| | r | p value | r | p value | r | p value | r | p value |
| | | | | | | | | |
| Fasting ISR (pmol/m2/min) | 0.09 | 0.30 | 0.00 | 0.93 | -0.00 | 0.95 | -0.06 | 0.52 |
| Total ISR (nmol/m2) | 0.04 | 0.66 | -0.05 | 0.59 | 0.07 | 0.47 | -0.06 | 0.52 |
| β-GS (pmol/m2/min/mmol) | -0.12 | 0.20 | 0.11 | 0.22 | -0.10 | 0.26 | -0.02 | 0.87 |
| Potentiation Factor (ratio) | -0.00 | 0.98 | 0.09 | 0.29 | -0.82 | 0.37 | 0.01 | 0.89 |
| Rate sensitivity (pmol/m2/mmol) | -0.14 | 0.13 | -0.02 | 0.80 | -0.01 | 0.94 | 0.02 | 0.87 |
